# Implementation of a Web-Based Work-Related Psychological Aftercare Program Into Clinical Routine: Results of a Longitudinal Observational Study

**DOI:** 10.2196/12285

**Published:** 2019-06-18

**Authors:** Rüdiger Zwerenz, Carlotta Baumgarten, Ingo Dahn, Nicole Labitzke, Andreas Schwarting, Matthias Rudolph, Peter Ferdinand, Ute Dederichs-Masius, Manfred E Beutel

**Affiliations:** 1 Department for Psychosomatic Medicine and Psychotherapy University Medical Center Johannes Gutenberg-University Mainz Germany; 2 Knowledge Media Institute University of Koblenz-Landau Koblenz Germany; 3 Center for Audiovisual Production Johannes Gutenberg-University Mainz Germany; 4 Center of Rheumatology University Medical Center Johannes Gutenberg-University Mainz Germany; 5 Mittelrhein-Klinik German Statutory Pension Insurance Rhineland-Palatinate Boppard, Bad Salzig Germany; 6 Drei-Burgen-Klinik German Statutory Pension Insurance Rhineland-Palatinate Bad Kreuznach Germany

**Keywords:** aftercare, internet, mental health, psychotherapeutic processes, return to work, occupational stress, health plan implementation

## Abstract

**Background:**

As inpatient medical rehabilitation serves to promote work ability, vocational reintegration is a crucial outcome. However, previous Web-based trials on coping with work-related stress have been limited to Web-based recruitment of study participants.

**Objective:**

The aim of our study was to evaluate the implementation of an empirically supported transdiagnostic psychodynamic Web-based aftercare program *GSA* (*Gesund und Stressfrei am Arbeitsplatz* [Healthy and stress-less at the workplace])-*Online plus* into the clinical routine of inpatient medical rehabilitation, to identify characteristics of patients who have received the recommendation for *GSA-Online plus*, and to determine helpfulness of the intervention and satisfaction of the participants as well as improvement in quality of life and mental health status of the regular users of *GSA-Online plus*.

**Methods:**

GSA-Online plus was prescribed by physicians at termination of orthopedic psychosomatic inpatient rehabilitation. Participants’ use of the program, work-related attitudes, distress, and quality of life were assessed on the Web.

**Results:**

In 2 rehabilitation centers, 4.4% (112/2562) of rehabilitants got a recommendation for *GSA-Online plus* during inpatient rehabilitation. Compared with usual person aftercare, the Web-based aftercare program was rarely recommended by physicians. Recommendations were made more frequently in psychosomatic (69/1172, 5.9%) than orthopedic (43/1389, 3.1%) rehabilitation (*χ*^*2*^_1_=11.845, *P*=.001, *Cramér V*=−0.068) and to younger patients (*P*=.004, *d*=0.28) with longer inpatient treatment duration (*P*<.001, *r*=−0.12) and extended sick leaves before inpatient medical rehabilitation (*P*=.004; *Cramér V*=0.072). Following recommendation, 77% (86/112) of rehabilitants participated in Web-based aftercare. Completers (50/86, 58%) reported statistically significant improvements between discharge of inpatient treatment and the end of the aftercare program for subjective work ability (*P*=.02, *d*=0.41), perceived stress (*P*=.01, *d*=−0.38), functioning (*P*=.002, *d*=−0.60), and life satisfaction (*P*=.008, *d*=0.42).

**Conclusions:**

Physicians’ recommendations of Web-based aftercare are well accepted by patients who derive considerable benefits from participation. However, a low rate of prescription compared with other usual aftercare options points to barriers among physicians to prescribing Web-based aftercare.

## Introduction

### Occupational Stress and Work-Related Medical Rehabilitation

Work-related stress, as observed in one-third of the German population [1], is an important risk factor for common mental disorders [2]. Conversely, mental disorders have become the leading reasons for long-term sickness absence [3] and premature pension in Germany [4]. The main purpose of inpatient medical rehabilitation is to restore or promote capacity to work of rehabilitants. Work-related medical rehabilitation (WMR) focusing on the workplace situation has been implemented successfully but needs to be complemented by work-related aftercare interventions to support rehabilitants during vocational reintegration [5]. However, evidence has been mixed. First, studies with intensified work-related orthopedic rehabilitation aftercare have not proven to be effective compared to aftercare without a focus on work-related topics [6]. On the contrary, participation in a graded return to work program reduced the relative risk of permanent work disability by about 40% as well as the time of welfare benefits owing to sickness absence compared with matched controls [7]. Yet, participation in aftercare programs following inpatient medical rehabilitation is low because of long waiting times, the lack of local aftercare providers, or incompatibility with family or work commitments [8,9].

Web-based interventions have been shown to be effective for a broad range of mental disorders, for example, depression [10], anxiety disorders [11], pain [12], substance abuse [13], and also improved physical activity [14] or a healthy diet and weight reduction [15] or psychosocial support for patients with chronic diseases [16]. The majority of the German working population (nearly 90%) is on the Web using the internet daily (72%) [17] and a substantial part is using it as a frequent source of health information (38%) [18]. Thus, Web- and mobile-based interventions may close gaps in routine care and improve diagnostics and treatment in medical rehabilitation [19] as widely accessible and cost-effective interventions [20].

### Web-Based Aftercare

Initial results of self-guided Web-based stress management interventions have been mixed. In a recent meta-analysis for Web-based interventions, moderate effect sizes were reported for overall 26 studies in reducing work-related stress (*d*=0.43, 95% CI 0.31-0.54) [21]. An internet- and mobile-based stress management program has proven effective in reducing perceived stress (*d*=0.96 posttreatment; *d*=0.65 6-month follow-up) compared with a waiting list control group and improved other relevant parameters of mental health, for example, depression, anxiety, and emotional exhaustion [22,23]. However, Web-based interventions when following inpatient medical rehabilitation are still rare [24] and interventions tested in randomized controlled trials have rarely been transferred into routine care.

We developed a Web-based transdiagnostic aftercare program (*GSA-Online*; *Gesund und Stressfrei am Arbeitsplatz* [*Healthy and stress-less at the workplace*]) that aimed at improving vocational reintegration of rehabilitants after long-term sickness absence. In our previous randomized controlled trial, *GSA-Online* had a statistically significant positive influence on the subjective prognosis of gainful employment (*d*=0.13 at the end of the intervention and *d*=0.20 at the follow-up 12 months after study inclusion). Furthermore we could show positive effects on affective mental distress (eg, *d*=0.25 for generalized anxiety or *d*=0.18 for depressive symptoms at follow-up) of the rehabilitants participating in the intervention [25] compared to the participants of an active control group.

### Study Aims and Research Question

The aims of this study were (1) to examine whether the further developed *GSA-Online plus* can be implemented into routine care of inpatient medical rehabilitation, (2) to identify characteristics of patients who have received the recommendation for *GSA-Online plus*, and (3) to determine perceived helpfulness of the intervention and satisfaction of the participants. With regard to our primary outcome, we hypothesized that (1) *GSA-Online plus* would be recommended as often as other established aftercare programs for rehabilitants with a special need for WMR-like psychological treatment, rehabilitation aftercare, or vocational rehabilitation; (2) at least 66% of the rehabilitants with a recommendation for *GSA-Online plus* would participate and write at least 1 diary entry after rehabilitation; and (3) completers, that is, regular users with at least 6 diary entries, would achieve (a) a more positive subjective prognosis of gainful employment, (b) an increased quality of life and lower perceived stress, and (c) an improvement of emotional distress such as depression and anxiety at the end of the intervention compared to discharge from rehabilitation.

## Methods

### Study Design

The study was conducted in 2 rehabilitation centers treating 3 different medical indications (psychosomatic and orthopedic rheumatic diseases). Inpatient medical rehabilitation entails a multimodal group-oriented approach, supplemented by individual therapy, addressing work ability of rehabilitants with chronic (>6 months), somatic, or psychological impairments [26]. In a pre-post design, data collection took place at 14 time points: after discharge from inpatient rehabilitation, that is, at the beginning (T1), once a week during (T2-T13; only active participants), and at the end of the aftercare intervention (T14).

Recruitment of the study was conducted for 10 months between June 2016 and March 2017. Inclusion criteria were (1) employment and a plan to return to work within 4 weeks after inpatient medical rehabilitation, (2) the ability to write in German language, (3) age between 18 and 59 years, and (4) a private internet access. During inpatient rehabilitation, the treating physician could prescribe the self-explanatory program by handing out an information leaflet to the patient and document it as a recommendation [27]. The recommendation was collected by the study assistant in each clinic who gave further study information, collected written consent, and gave access data for *GSA-Online plus* to the participant.

#### Intervention

The intervention *GSA-Online plus* aimed to support occupationally stressed rehabilitants during their return to work. In a structured psychodynamic format [28], participants were instructed to identify and articulate interpersonal and intrapsychic problems during return to work. Participants got weekly personalized writing impulses from a trained and supervised therapist to help them write in the form of a diary about their experiences of returning to their workplace. Therapeutic commentaries (usually within 24 hours) to their Web-based diary entry processed interpersonal and intrapsychic problems and helped participants deal with their individual job-related problems and stabilize their working capacity. Usually, it took 20 to 40 min per week per participant for the therapist to write an answer to the diary entry and add another writing impulse. In addition to the previous version (*GSA-Online*), educational video clips were used to familiarize participants with the program and its features (see Multimedia Appendices 1-4). For a detailed description of the therapeutic rationale, see Beutel et al [28].

#### Measures

As primary outcomes, the medical referral rate was documented (frequency of recommendations of *GSA-Online plus*) and participants’ utilization of *GSA-Online plus* was tracked with PIWIK (now Matomo [29]), a secure open Web analytics platform and assessed with self-constructed single items (eg, “Please indicate how often you have used *GSA-Online plus* since the end of your inpatient treatment.”).

In pre- and postmeasurements, the secondary outcome subjective prognosis of gainful employment was assessed with the SPE-Scale [30] consisting of 3 items assessing a subjective rating of future employment until retirement age, the impairment of work ability by the current health status, as well as the plan to apply for a premature pension. The 3 items could be added up to a score between 0 and 3 with a higher score indicating a higher risk for premature pension. The capacity to work was assessed with the short form of the work ability index (WAI), a 7-item scale with a reliability of alpha=.78 in a German population (eg, “How do you estimate your current work ability in terms of physical work requirements?”) [31]. Mental disorders were assessed with different subscales of the German version of the Patient Health Questionnaire (PHQ-D) by Löwe et al [32]. Depressive symptoms were assessed with the 9-item scale PHQ-9 (eg, “Over the last two weeks, how often have you been bothered by little interest and pleasure in doing things?”) [33], with an internal consistency of alpha=.89. Stress symptoms were assessed with the 10-item stress module PHQ-Stress (eg, “Over the last four weeks, how often have you been bothered by worries about health?” *)* [34,35]. Anxiety symptoms were assessed with the 7-item scale for General Anxiety Disorder (GAD-7; eg, “Over the last 2 weeks, how often have you been bothered by feeling nervous, anxious or on edge?” *,* alpha=.92) [36]. Somatoform symptoms were assessed with the 8 items of the Somatic Symptom Scale-8 (eg, “During the past 7 days, how much have you been bothered by stomach or bowel problems?”, alpha=.81) [37] also based on the PHQ. Psychosocial stressors were measured with the 4-item short form of the Perceived Stress Scale (PSS-4; eg, “In the last month how often have you felt you were unable to control the important things in your life?”, alpha=.60-.82) [38]. General functioning was measured with the 3-item Sheehan Disability Scale (eg, “To what extent do your symptoms impair your functioning in your social life?”, alpha=.89) [39,40], where each item can be scored from 0 to 10 resulting in a global score from 0 (unimpaired) to 30 (highly impaired). Resources were assessed with the 3-item Oslo Social Support Scale (eg, “How many people are so close to you that you can count on them if you have great personal problems?”, alpha=.60) [41], with the 4-item Brief Resilient Coping Scale (eg, “I look for creative ways to alter difficult situations”, internal consistency *r*=0.76) [42], with the Loneliness Scale [43] and with the questions on life satisfaction, a scale that consists of 2 8-item modules (general life satisfaction and satisfaction with health, alpha=.82-89) [44]. Patient satisfaction was assessed with the 8-item Client Satisfaction Questionnaire (eg, “How would you rate the quality of the service you have received?”, alpha=.93) [45]. In a weekly query, participants were asked with 2 items on a 5-point Likert scale (from 0=not at all to 4=very) about their satisfaction with *GSA-Online plus* (“How satisfied are you with the feedback of the online therapist?” and “How helpful was the feedback of the online therapist?”) and rated their overall health condition with 1 item of the German version of the EuroQol Questionnaire (EQ-5D) [46] (“Your own health status today”) as well as their current work ability with the first item from the WAI [31] also known as the Work Ability Score (WAS) [47], each on a Likert scale from 0 to 10. At the end of the aftercare program, participants were asked if and how much they would pay for *GSA-Online plus* with self-constructed items.

All questionnaires were assessed with the Web-based survey platform, SoSci Survey [48], except the weekly assessments that have been directly implemented in the platform of *GSA-Online plus*.

#### Statistical Analyses

All statistical analyses were done with IBM SPSS Statistics 23 [49]. Recommendation rates and utilization of *GSA-Online plus* were analyzed with cross-sectional analyses and participants of the Web-based aftercare were compared with the population of all rehabilitants treated during the recruitment period, using descriptive statistics (chi-square tests and *t* tests, Mann-Whitney U tests if the required assumptions for parametric testing, for example, homogeneity of variance, were violated). Pre-post changes were analyzed with per protocol data as secondary outcomes with longitudinal data analysis (*t* tests, rmANOVA, and descriptive statistics). To estimate treatment effects, Cohen *d* was calculated for the comparison of mean scores with *t* tests, Cramér V for chi-square tests and the effect size r was calculated for the comparison of median scores with the Mann-Whitney U test.

For the weekly assessment of the general health status and the subjectively rated ability to work, missing data were replaced by the last observation carried forward (LOCF) procedure. To analyze improvement across time, a repeated measures analysis of variance was conducted.

#### Ethics Approval

The study protocol was approved by the Ethics Committee of the Federal State of the Rhineland Palatinate (Approval Number: 837.175.16(10494)).

## Results

### Recommendation Rates for *GSA-Online plus*

Of the 2562 rehabilitants in 2 rehabilitation centers, 112 (4.4%) got a recommendation for *GSA-Online plus* during inpatient rehabilitation, which was significantly lower than the referral rate to other face-to face aftercare interventions (Table 1). Recommendation rates were higher in psychosomatic rehabilitation, where psychological treatments were routinely recommended in 88% (1030/1147) versus 8% (117/1147) after orthopedic rehabilitation. Rehabilitation aftercare was more frequently prescribed in orthopedic (500/1389, 36%) than in psychosomatic rehabilitation (89/1172, 8%). A total of 11% (291/2561) received recommendations of vocational rehabilitation.

**Table 1:** Recommendation rates at discharge for aftercare in total and in the 2 rehabilitation centers.

Aftercare recommendation	Psychosomatic rehabilitation center (n=1172), n (%)	Orthopedic and rheumatoid rehabilitation center (n=1389), n (%)	Total (N=2561), n (%)	χ^2^_1_	*P* value	*d*	*Cramér V*
*GSA-Online plus*	69 (5.9)	43 (3.1)	112 (4.4)	11.845	.001	0.14	−0.068
Psychological Treatment	1030 (87.9)	117 (8.4)	1147 (44.8)	1623.45	<.001	2.63	−0.796
Rehabilitation aftercare	89 (7.6)	500 (36.0)	589 (23.0)	289.57	<.001	0.71	0.336
Vocational rehabilitation	123 (10.5)	168 (12.1)	291 (11.4)	1.616	.20	0.05	0.025

### Patients’ Characteristics Associated With *GSA-Online plus* Recommendation

Rehabilitants who were recommended *GSA-Online plus* were younger (mean 47.67 [SD 9.97] vs mean 50.31 [SD 9.5], *t*_2559_=2.865, *P*=.004, *d*=0.28) and had longer rehabilitation treatments (median 35 days vs median 28 days, *U*=182642.00, *P*<.001, *r*=−0.12). Furthermore, they reported longer work disability before inpatient rehabilitation (*P*=.004), and were more often (marginally significant, *P*=.07) considered able to work at discharge than rehabilitants who did not receive a recommendation (Table 2).

Of the 112 rehabilitants with a recommendation for *GSA-Online plus*, 77% (86/112) gave written informed consent and registered on the Web to participate in *GSA-Online plus*. From these, 58.1% (50/86) wrote at least 6 (completers) and 41.9% (36/86) less than 6 diary entries (dropouts). Multimedia Appendix 5 shows the distribution of the number of diary entries. Dropouts wrote an average of almost 3 diary entries (mean 1.53, [SD 1.72]), completers wrote 10 diary entries (mean 10.34 [SD 2.00]; *t*_84_ =−21.4, *P*<.001, *d*=−4.68). Two-thirds of completers (66.0%) wrote 11 or 12 diary entries.

Completers were older (mean 49.36 [SD 9.04] vs mean 45.14 [SD 10.49], *t*_84_=−1.998, *P*=.049, *d*=0.44) and more often female (74.5% vs 50%, *χ*^*2*^_*1*_=4.638, *P*=.003) than dropouts. Further sociodemographic variables revealed no significant differences between completers and dropouts.

**Table 2:** Sociodemographic characteristics (T1: baseline): comparison of rehabilitants with and without recommendation for *GSA-Online plus*.

Sociodemographic characteristics		With recommendation (n=112), n (%)	Without recommendation (n=2449), n (%)	Total (N=2561), n (%)
**Sex^a^**				
	Male	45 (40.2)	1067 (43.6)	1112 (43.4)
	Female	67 (59.8)	1382 (56.4)	1449 (56.6)
**Marital status^b^**				
	Partnership	60 (53.6)	1525 (62.3)	1585 (61.9)
	No partnership	47 (42.0)	768 (31.4)	815 (31.8)
	Unknown	5 (4.5)	156 (6.4)	161 (6.3)
**Inability to work before inpatient admission^c^**				
	No inability or not employed	11 (9.8)	347 (14.2)	358 (14.0)
	3 months or less	52 (46.4)	1061 (43.3)	1113 (43.5)
	3 to 6 months	29 (25.9)	370 (15.1)	399 (15.6)
	6 months or more	20 (17.9)	671 (27.4)	691 (27.0)
**Work ability at discharge^d^**				
	Able to work	67 (59.8)	1203 (49.1)	1270 (49.6)
	Unable to work	44 (39.3)	1196 (48.8)	1240 (48.4)
	Unknown	1 (0.9)	50 (2.0)	51 (2.0)

^a^*χ*^2^_1_=0.501, *P*=.50, *Cramér V*=0.014.

^b^*χ*^2^_2_=5.713, *P*=.06, *Cramérs V*=0.047.

^c^*χ*^2^_3_=13.295, *P*=.004, *Cramérs V*=0.072.

^d^*χ*^2^_2_=5.20, *P*=.07, *Cramérs V*=0.045.

### Effectiveness of *GSA-Online plus* and Participants’ Satisfaction

**Figure 1:**
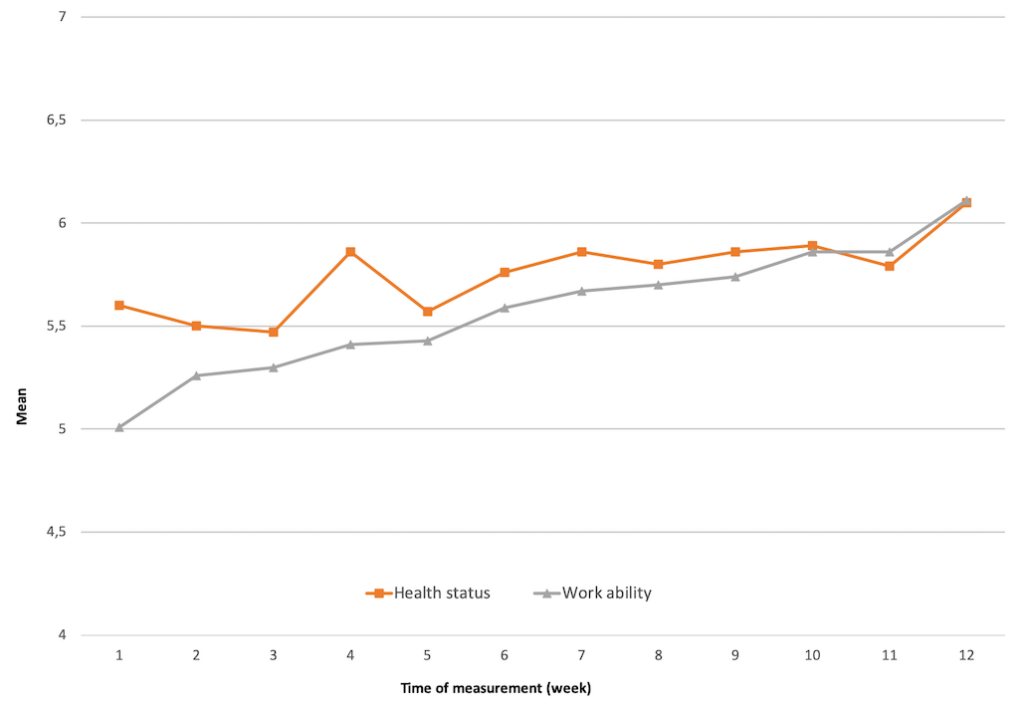
Means of the subjective health status and subjective work ability, assessed in the course of the aftercare from T2 to T13. Subjective health status was assessed with the EuroQoL-5D single item “How good or bad is your health status today?” 0=worst imaginable health; 10=best imaginable health. Subjective work ability was assessed with the Work Ability Score “Current ability to work in comparison with the best, ever reached ability to work”; (range 0-10). N=70, missing data replaced with last observation carried forward.

In Figure 1 the weekly ratings of work ability and subjective health status are displayed. Missing data were replaced with the last observation carried forward method.

A statistically significant improvement could be observed for the subjective rated work ability assessed with the WAS (*F*_11,759_=3.808, *P*<.001) but not for the subjective health status assessed with 1 item of the EQ-5D (*F*_11,759_=3.928, *P*=.22).

Satisfaction with *GSA-Online plus* was generally high. 43.5% (20/46) were largely and 45.7% (21/46) were very satisfied with *GSA-Online plus* overall and the level of support was also rated very positive. In total, 47.8% (22/46) were largely and 32.6% (15/46) were very satisfied with the help they got.

As displayed in Multimedia Appendix 6, for the weekly monitoring of satisfaction and rated helpfulness of the therapeutic feedback, a repeated measures analysis of variance was calculated. A statistically significant improvement (Multimedia Appendix 6) could be observed for the satisfaction with the feedback from a Web-based therapist (*F*_11,176_=2.005, *P*=.03) as well as for the subjectively rated helpfulness (*F*_11,176_=2.919, *P*=.001)—both assessed weekly with 1 item on a Likert scale from 0 to 4. For the purpose of sensitivity analysis, data of participants (N=17) who wrote 12 diary entries were analyzed separately leading to a clear increase of satisfaction and helpfulness (Multimedia Appendix 7).

Table 3 shows secondary outcome measures for regular users of *GSA-Online plus* (completers). Statistically significant improvements could be observed between discharge from the rehabilitation center and the end of the aftercare program (ie, 3 months after discharge) for subjective work ability as assessed by the WAI (*P*=.02, *d*=0.41), perceived stress assessed with the PSS-4 scale (*P*=.01, *d*=−0.38), functioning assessed with the SDS (*P*=.002, *d*=−0.60), and life satisfaction (*P*=.008, *d*=0.42). The other symptom scales scores remained stable and did not change between discharge and the follow-up assessment.

**Table 3:** Secondary outcome measures of completers of *GSA-Online plus* at discharge from inpatient medical rehabilitation (T1) and at the end of aftercare (T14).

Secondary outcomes	T1^a^, mean (SD)	T14^b^, mean (SD)	*P* value^c^	Effect size (*d*)^d^
Prognosis of gainful employment (n=44)	0.909 (1.007)	0.932 (0.998)	.82	0.03
Work ability (n=44)	25.080 (5.968)	27.125 (7.030)	.*02*^e^	0.41
Depression (n=46)	9.565 (4.420)	8.870 (4.400)	.17	−0.21
Anxiety (n=46)	7.348 (4.132)	7.587 (4.203)	.66	0.07
Somatic symptom (n=45)	11.667 (6.142)	11.733 (5.618)	.90	0.02
Psychosocial stress (n=46)	7.396 (3.655)	7.913 (3.601)	.26	0.17
Perceived stress (n=44)	7.955 (1.976)	7.136 (2.339)	*.01* ^e^	−0.38
General functioning (n=44)	22.386 (5.406)	18.318 (7.398)	*.002* ^f^	−0.60
Loneliness (n=44)	7.727 (3.022)	7.144 (3.166)	.46	−0.27
Social support (n=44)	9.272 (2.386)	9.386 (2.315)	.60	0.08
Coping (n=43)	14.047 (3.280)	14.907 (3.069)	.09	0.16
Life satisfaction (n=43)	25.581 (5.261)	26.814 (5.193)	*.008* ^f^	0.42

^a^Baseline.

^b^End of intervention.

^c^*t*-test for dependent samples; statistically significant differences are italicized.

^d^*d*: Cohen *d*.

^e^Significant at *P*<.05.

^f^Significant at *P*<.01.

Of the 46 participants who completed the Web-based questionnaire at measurement T14, the majority (27/46, 58.7%) watched at least one of the educational video clips but also almost half (19/46, 41.3%) had not seen any of the films. The main reasons for not watching any films were that the existence of the films was unknown to the participants (8/19, 42%), participants did not use any other features of *GSA-Online plus* besides the Web-based diary (5/19, 26%) or reported a lack of time (5/19, 26%). In total, 77% (23/30) of the participants who answered questions about video use assessed the films as a positive contribution to the comprehensibility of *GSA-Online plus*.

Finally, the willingness of participants to pay for *GSA Online Plus* was assessed at T14. Participants were asked if they would be willing to pay *for GSA-Online plus* and how much they would pay if they were required to pay. Of the 46 participants who answered the first question, 37% (17/46) said they were willing to pay for *GSA-Online plus* and 40 participants reported to pay in average (mean) 174.25 Euro (SD 292.2, Min 0, Max 1500).

## Discussion

### Principal Findings

Inpatient medical rehabilitation serves to promote work ability and vocational reintegration is a crucial outcome. Although previous Web-based trials have improved coping with work-related stress [23] or showed that Web-based aftercare could successfully maintain effects of inpatient treatment [50], information on implementation processes are missing because previous Web-based interventions have been conducted under study conditions, and participants were usually recruited on the Web. The purpose of the trial was to evaluate the implementation of a Web-based aftercare program (*GSA-Online plus*) during inpatient medical rehabilitation.

As we had hypothesized, it was feasible to implement the program, and the great majority who received the recommendation (86/112, 77%) actually logged into the program, slightly exceeding our expectations. Among those who started writing blogs, subjective or perceived rated work ability increased over the course of their participation. Satisfaction with Web-based aftercare was generally high. Acceptance was good with 43.5% (20/46) of the participants being largely and 45.7% (21/46) very satisfied with *GSA-Online plus* overall and the level of support was also rated very positively with 47.8% (22/46) of the participants being largely and 32.6% (15/46) very satisfied with the help they received. Contrary to our hypotheses, subjective health status did not increase significantly. However, there was a significant increase of subjective work ability, general functioning, as well as life satisfaction and a decrease of subjective stress. Especially, work ability and functioning are major issues in rehabilitation; therefore, a stabilization of these factors is a good indicator that our aftercare intervention had a focus on the right topics. Unfortunately, no change was found regarding mental distress for the completers of the intervention with a per protocol analysis. An explanation could be, that mental distress was not so high in this sample, with not only psychiatric but also orthopedic main diagnoses, therefore, an improvement was hard to detect in the relatively small sample we could include in our pre-post analyses.

However, unexpectedly, the referral rate by the physicians of about 4% was substantially lower than all face-to-face aftercare offers and there was also a lot of individual variation among physicians. Recommendations were made more frequently in psychosomatic (5.9% [69/1172]) than orthopedic rehabilitation (3.1% [43/1389]) and more often to younger patients with extended sick leaves and lengthy inpatient stays. This finding coincides with the results of Hennemann et al [51], showing clear preconceptions and barriers regarding Web-based contact among rehabilitation staff, concerning disruption of face-to-face therapeutic alliance and a lack of sufficient data security in Web-based aftercare interventions.

Owing to the low proportion of recommendations, we asked members of the treatment team of the rehabilitation centers in a Web-based survey at the end of the study for their subjective criteria and frequencies of recommendation of *GSA-Online plus*. Unfortunately, only a few members (N=19) of the treatment team took part in this assessment and the biggest part (8/19, 61.5%) made less than 10 referrals for *GSA-Online plus*, whereas 23.1% (3/19) referred 11 to 20 and 15.4% (2/19) more than 20 rehabilitants. Members of the treatment team were informed about *GSA-Online plus* in a one-time on-site training and an informational paper that was accessible to all throughout the study. One explanation for a different referral rate between different team members could well be that especially those who did not attend the training did not make so many recommendations. Data we have on this point at least suggest that there was a trend that referral rate of those who did not attend the training was somehow lower than of those who attended the training. Asked, whether Web-based interventions could be a useful supplement or substitute for regular face-to-face interventions the majority (8/19, 42.1%) answered that they could be a useful supplement but no one rated them as a substitute to usual care.

Our finding is of special interest, because the 121st German congress of physicians has recently lifted the ban on remote treatment [52]. Furthermore, the German statutory pension insurance scheme recently developed requirements for the implementation of tele medical aftercare programs into routine care [53].

Previously, reservations towards occupational e-mental health interventions were also found in patients, especially in the risk groups the interventions were planned for [54]. In spite of the low recommendation rate, the intervention has shown promise in participants. Thus, physicians are in a prime position to provide access to and motivate their patients for sustained participation in these programs. Our findings implicate that 2 steps should be pursued in the future: (1) Awareness about the benefits of Web-based interventions in routine care and their compatibility as an adjunct to face-to face aftercare are required to promote openness in the treatment team to recommend, and to increase referral rates for innovative interventions. (2) Physicians need to be instructed about their patients’ reservations to sign up for Web-based support and enabled to help their patients initiate and maintain their engagement in suitable Web-based programs.

### Limitations

Limitations of our study were that we had no control group to analyze efficacy in a routine care setting and that we did not reach our anticipated sample size, because of the much lower referral rate than expected. It also may be debated if logging in once is a sufficient criterion for participation.

The LOCF method for imputing missing data is not the gold standard but seemed sufficient in this small sample, especially as efficacy only was a secondary research question. Furthermore, the multiple comparisons within our secondary research questions are to be considered as a limitation. After adjustment for multiple testing, only general functioning will remain as a significant improvement after participating in *GSA-Online plus*.

Unfortunately, we did not ask participants about advantages of Web-based interventions but we asked them about their digital competencies. Two-thirds of the participants rated their knowledge of using digital media (eg, PC, smartphone, and tablet) as mediocre to rather good. Overall, the attitude toward the internet was positive, but one-third also stated that they feel burdened by the constant availability via mobile phone or email.

Hence, future implementations should focus more on collaborating with staff and clinicians in rehabilitation clinics to address potential prejudices and barriers to Web-based aftercare. On the patient side, it is important to address advantages and disadvantages of Web-based interventions to improve acceptance for internet- and mobile-based interventions.

### Conclusions

Web-based psychological aftercare proved to be effective to reduce treatment gaps after inpatient medical rehabilitation but only for a limited number of rehabilitants. *GSA-Online plus* has been provided as a Web-based aftercare that offers all inpatient medical rehabilitation patients with occupational exposure, mental comorbidity, and an intentional timely return to work the opportunity to be promoted and assisted with professional reintegration by trained psychologists. Once a recommendation for *GSA-Online plus* was given from the physician in routine care, it could lead to a significantly higher participant motivation and adherence than in a controlled efficacy study. Outcome criteria on which the Web-based aftercare focuses (ability to function, work ability, general state of health, and life satisfaction) will improve even further in the course of follow-up, compared with the state of health at the end of inpatient rehabilitation. Motivation of rehabilitants and attitudes of the treatment team toward Web-based interventions are essential to improve implementation/recommendation rates. An important question for future research could be how Web-based interventions for rehabilitants with work-related problems could be optimized or supplemented to reach more rehabilitants. As the return to work is a major issue in rehabilitative treatment in Germany, a more practical oriented intervention with a more social work-driven focus could possibly close this gap and continue the multidisciplinary treatment approach that is already one main characteristic of inpatient medical rehabilitation in Germany.

## Appendix

Multimedia Appendix 1 Screenshot of the landing page.

Multimedia Appendix 2 Overview of the online diary.

Multimedia Appendix 3 Screenshot of an explanatory video.

Multimedia Appendix 4 Screenshot of an explanatory video.

Multimedia Appendix 5 Number of written online diary entries (completer vs dropouts).

Multimedia Appendix 6 Means of the weekly monitoring of satisfaction and rated helpfulness of the therapeutic feedback, both assessed weekly with one item each on a Likert-scale from 0 to 4. N=70, missing data replaced with last observation carried forward.

Multimedia Appendix 7 Means of the weekly monitoring of satisfaction and rated helpfulness of the therapeutic feedback, both assessed weekly with one item each on a Likert-scale from 0 to 4. N=17, per protocol data.

## Abbreviations

EQ-5D: EuroQol questionnaire
GSA: Gesund und Stressfrei am Arbeitsplatz [Healthy and stress-less at the workplace]
LOCF: Last observation carried forward
PHQ: Patient Health Questionnaire
PSS-4: Perceived Stress Scale
WAI: Work ability index
WAS: Work Ability Score
WMR: Work-related medical rehabilitation
