# Supplementary material for: Implementation of a Web-Based Work-Related Psychological Aftercare Program Into Clinical Routine: Results of a Longitudinal Observational Study
Source: J Med Internet Res. 2019 Jun 18;21(6):e12285. doi: 10.2196/12285 (PMC6604507; doi:10.2196/12285)
Supplement: Multimedia Appendix 1 [file jmir_v21i6e12285_app1.pdf]

GSA+online

Gesund und stressfrei am Arbeitsplatz

UNIVERSITÄTSmedizin. MAINZ

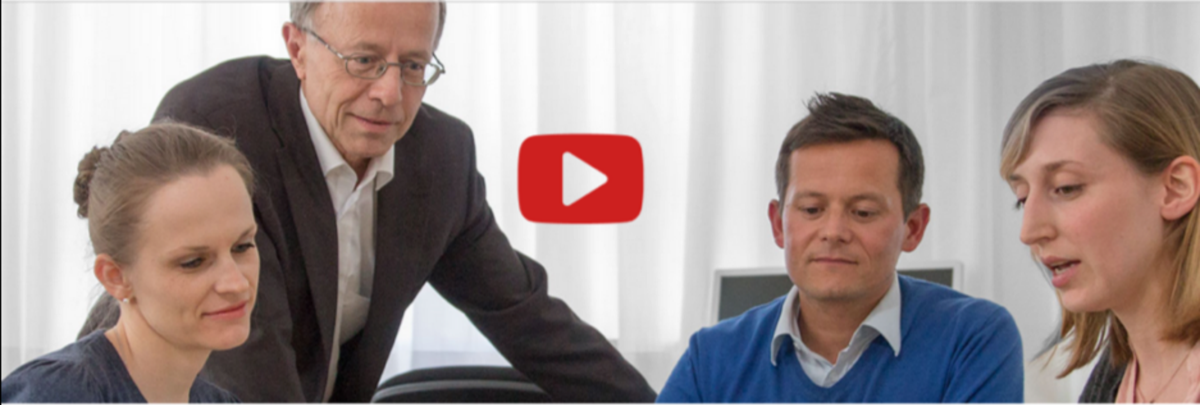

Registrierung

Persönlicher Code

Code

zur Registrierung

Login

Benutzername

Benutzername

Passwort

Passwort

Eingeloggt bleiben

Login Passwort vergessen?
