# Supplementary material for: Implementation of a Web-Based Work-Related Psychological Aftercare Program Into Clinical Routine: Results of a Longitudinal Observational Study
Source: J Med Internet Res. 2019 Jun 18;21(6):e12285. doi: 10.2196/12285 (PMC6604507; doi:10.2196/12285)
Supplement: Multimedia Appendix 2 [file jmir_v21i6e12285_app2.pdf]

GSA+online
Gesund und stressfrei am Arbeitsplatz

Mein Tagebuch   Info-Videos   carson.toy

Tagebuch ?

Dies ist Ihr Tagebuch. Es enthält eine Übersicht aller geplanten und geschriebenen Einträge mit ihrem jeweiligen Status.

Es ist Woche **12** von 12.

Klicken Sie auf die jeweilige Woche, um diese auszuklappen.
Zum Eintrag gelangen Sie über den Link auf der rechten Seite der Zeile.

› **Woche 1** - Tagebucheintrag und Rückmeldung abgeschlossen
Zum Eintrag ›

› **Woche 2** - Tagebucheintrag und Rückmeldung abgeschlossen
Zum Eintrag ›

› **Woche 3** - Bearbeitungszeit beendet
Zum Eintrag ›

› **Woche 4** - Bearbeitungszeit beendet
Zum Eintrag ›

› **Woche 5** - Ihr Onlinetherapeut hat geantwortet - bitte bewerten Sie seine Rückmeldung.
Zum Eintrag ›
