# Supplementary material for: Implementation of a Web-Based Work-Related Psychological Aftercare Program Into Clinical Routine: Results of a Longitudinal Observational Study
Source: J Med Internet Res. 2019 Jun 18;21(6):e12285. doi: 10.2196/12285 (PMC6604507; doi:10.2196/12285)

Rückkehr an den Arbeitsplatz

Warum kann eine Online-Nachsorge beim beruflichen Wiedereinstieg hilfreich sein?

zum Seitenanfang

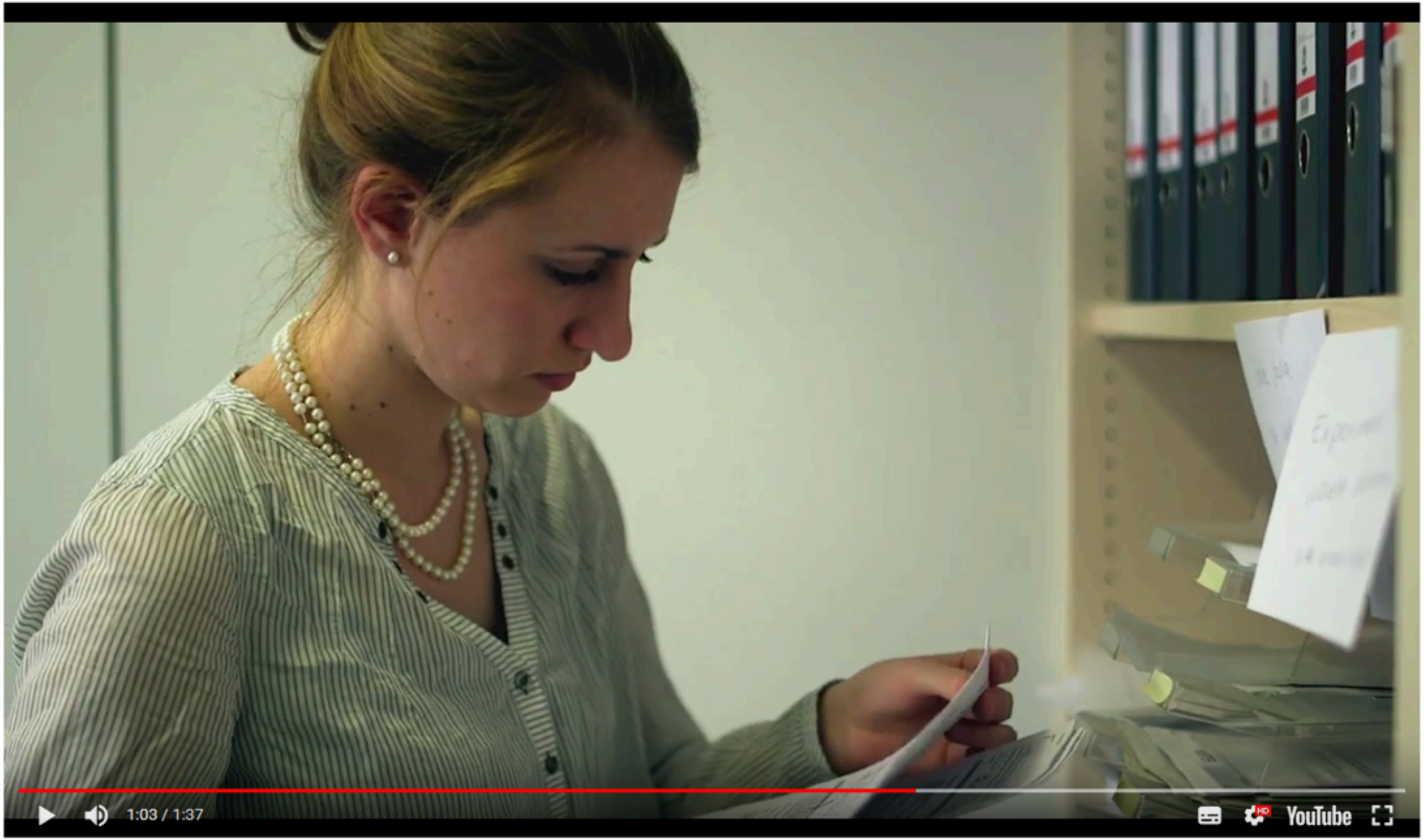

Supplement: Multimedia Appendix 3 [file jmir_v21i6e12285_app3.pdf]
