# Supplementary figures and images for: Implementation of a Web-Based Work-Related Psychological Aftercare Program Into Clinical Routine: Results of a Longitudinal Observational Study
Source: J Med Internet Res. 2019 Jun 18;21(6):e12285. doi: 10.2196/12285 (PMC6604507; doi:10.2196/12285)

# Ein Beispiel

Wie kann die Rückkehr an den Arbeitsplatz ablaufen?

[↑ zum Seitenanfang](#)

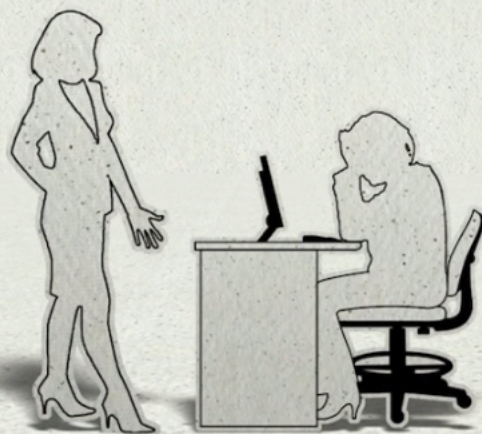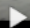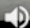

1:15 / 3:09

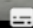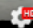

YouTube

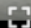

Supplement: Multimedia Appendix 4 [file jmir_v21i6e12285_app4.pdf]

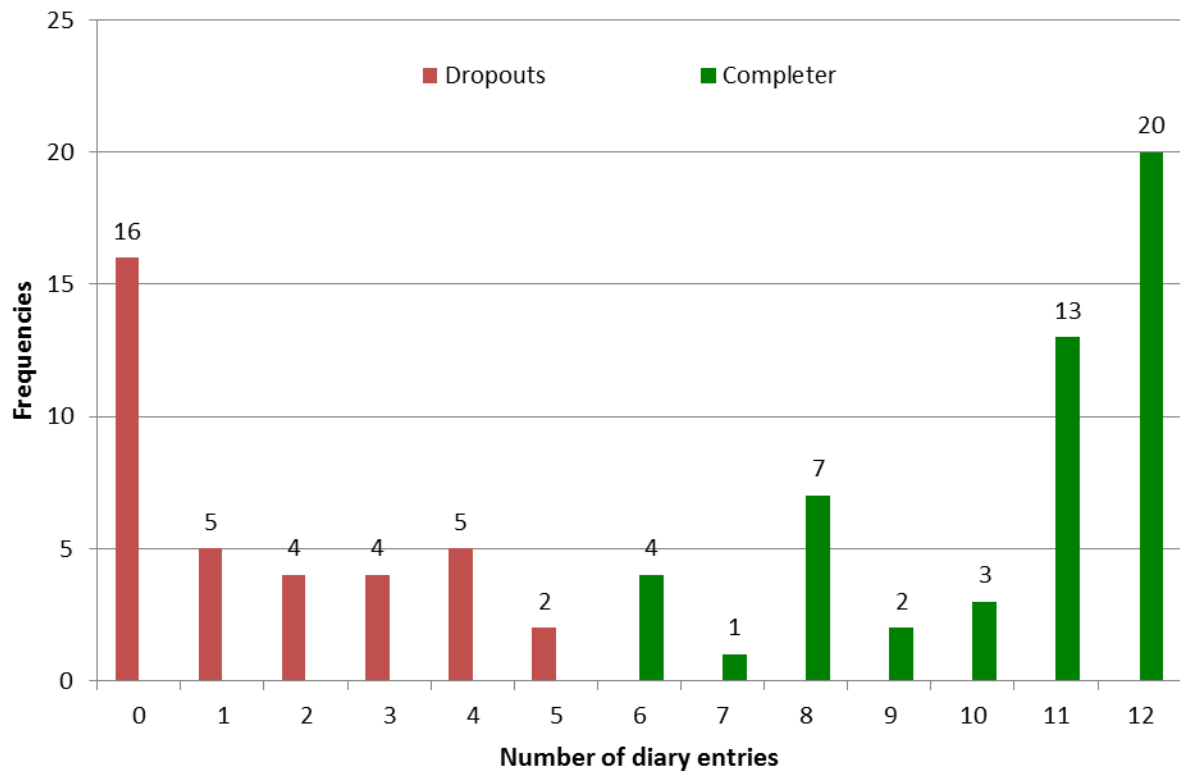

Supplement: Multimedia Appendix 5 [file jmir_v21i6e12285_app5.pdf]

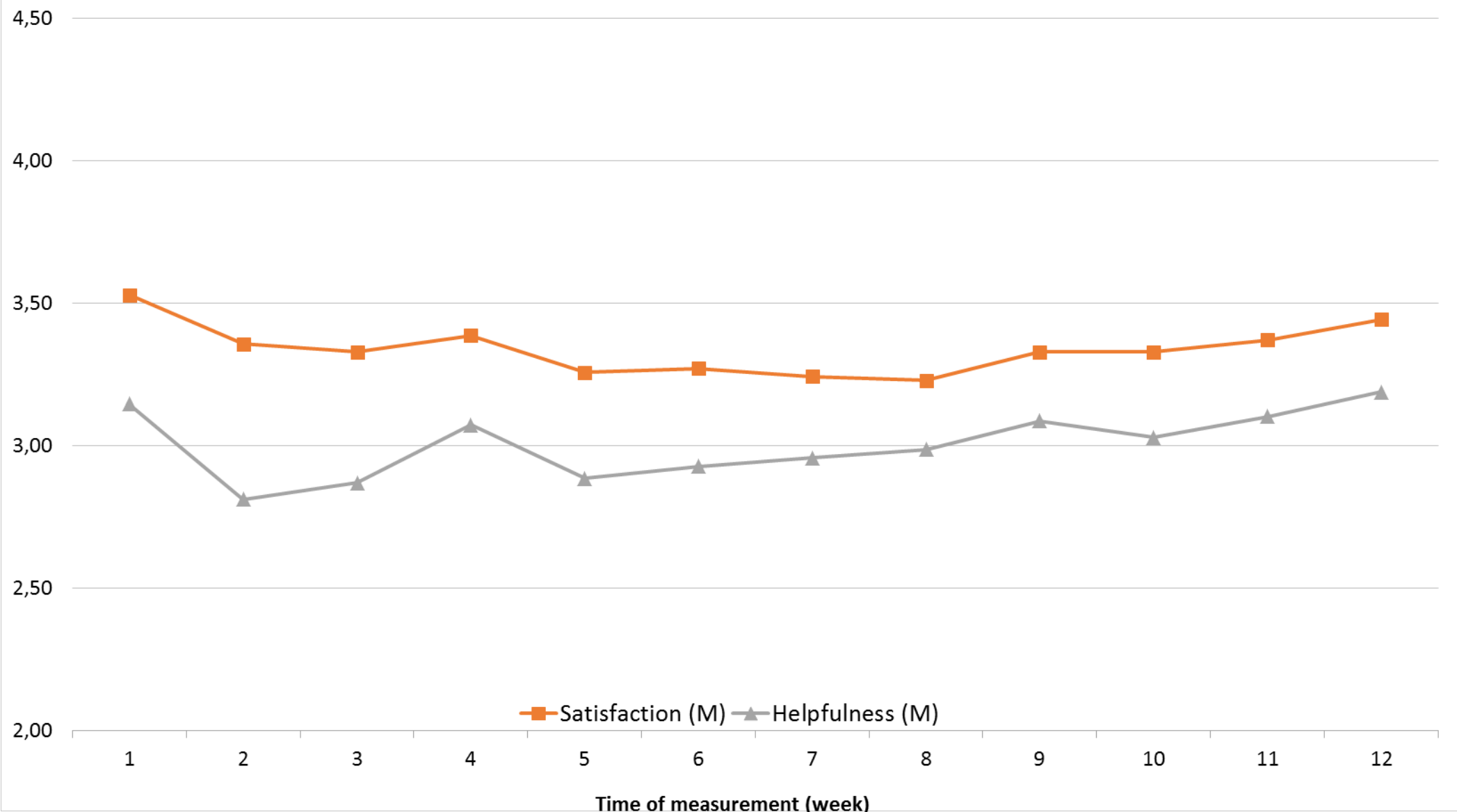

Supplement: Multimedia Appendix 6 [file jmir_v21i6e12285_app6.pdf]

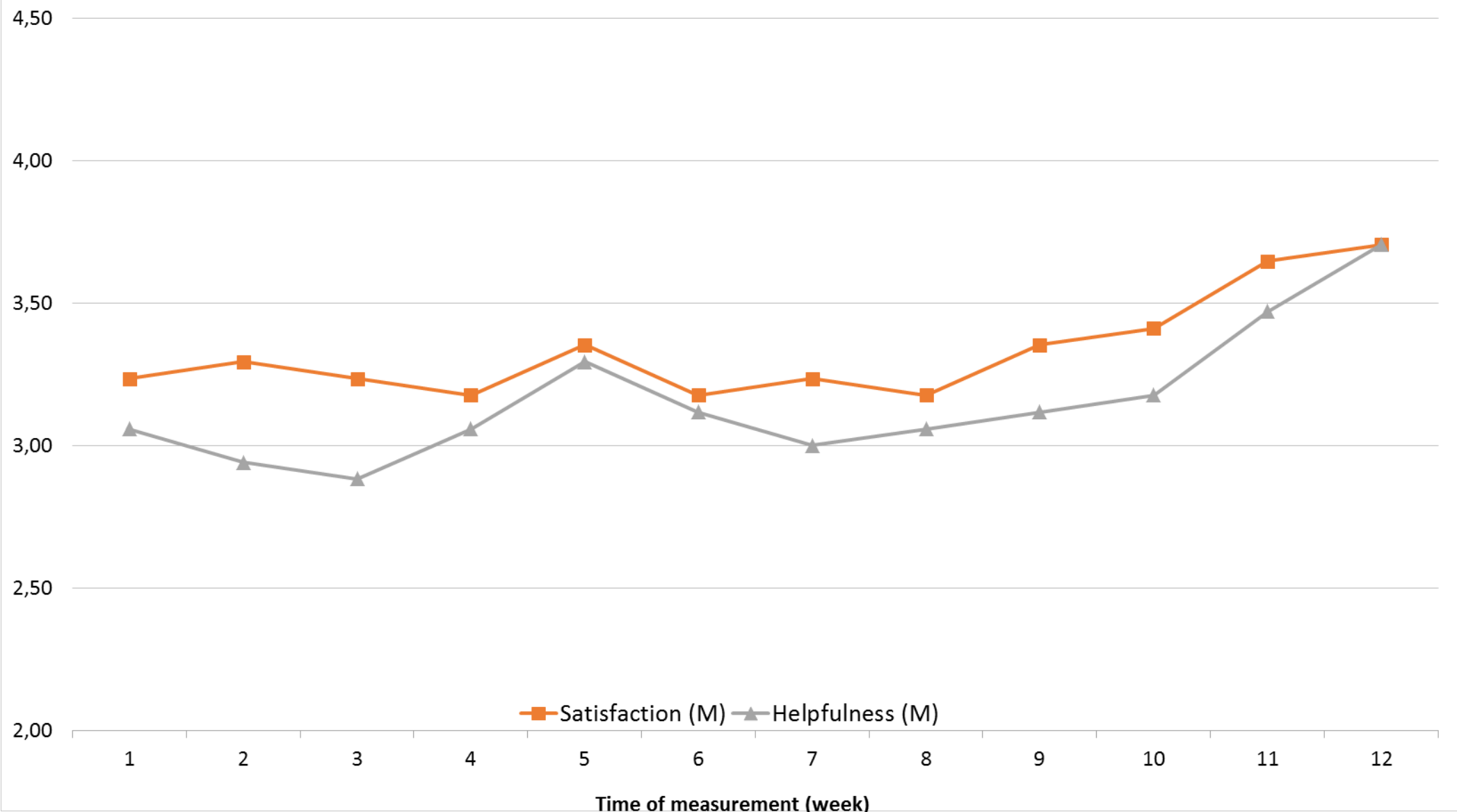

Supplement: Multimedia Appendix 7 [file jmir_v21i6e12285_app7.pdf]
